# The experience of general practitioners with Very Brief Advice in the treatment of tobacco addiction

**DOI:** 10.1038/s41533-020-00200-0

**Published:** 2020-09-23

**Authors:** Onno C. P. van Schayck, Lynn Bindels, Ancka Nijs, Bo van Engelen, Adrienne van den Bosch, Ilona Statius Muller, Mark Spigt

**Affiliations:** 1 Department of General Practice CAPHRI Care and Public Health Research Institute, Maastricht University, Maastricht, the Netherlands; 2 Zorroo, Zorggroep Regio Oosterhout & Omstreken, Oosterhout, the Netherlands; 3 Ubbens & Statius Muller Huisartsen, Amsterdam, the Netherlands

**Keywords:** Lifestyle modification, Preventive medicine

## Abstract

Although tobacco smoking is the world’s most important preventable cause of many chronic diseases (including COPD and asthma) and premature death, many physicians do not routinely apply smoking cessation in the daily health care of their patients. Two widely felt important concerns of physicians are that smoking cessation as part of a treatment is time-consuming and may jeopardize their relationship with patients. Very Brief Advice (VBA) is a non-confrontational method, which could assist general practitioners (GPs) as a simple, quick first step in getting patients to stop smoking. In this study, we investigated the opinions and experiences of GPs with VBA in their routine care in two rounds of telephone interviews with 19 GPs. The interviews were recorded and transcribed and subsequently analysed with NVivo12. We observed that the GPs had a very positive experience with using VBA. They found the method to be efficient as to the time involved, patient-friendly and easy to implement.

## Introduction

Tobacco smoking remains one of the main preventable causes of chronic diseases (including asthma and chronic obstructive pulmonary disease) and premature death worldwide^1,2^. Nicotine from cigarettes generates a strong urge to smoke, while suppressing any rational concerns about the negative consequences of smoking, and as a consequence the failure of the stop attempt for those trying to quit^1^.

General practitioners (GPs) often do not routinely provide evidence-based smoking cessation treatment. Research shows that many GPs hesitate to discuss the topic of smoking cessation with their patients^3^. Studies show a few reasons for this phenomenon, with the GPs perceiving a lack of time to discuss the topic, lacking communication skills, not wanting to jeopardize the relationship with their patient, feeling frustrated with patients who continue to smoke despite the fact that they are ill due to smoking, or not feeling competent to discuss this subject^4,5^.

Very Brief Advice (VBA) is supposed to support all smokers, regardless of their present motivation to quit. It is a non-confrontational method taking <30 s and can be used by any health-care practitioner after some training^6^. Moreover, this method proves to be very effective as a first step in the process of actually stopping smoking^7–9^. If VBA were to be widely used in general practice, it is expected that many more smokers would probably attempt to stop smoking. Although exact data on the actual use of VBA by GPs are not available, it is widely recognized that VBA is not generally applied in routine care^2^.

The present study investigates the effects of VBA in Dutch general practice. In this study, we focussed on GPs, and not on nurse practitioners or assistants, because VBA given by GPs appears to be effective, possibly because of a so-called ‘white coat’ effect^10,11^.

Figuring out what GPs who do not use VBA think about this new approach could bridge the gap between the effects in research settings and routine care. The main purpose of this study is therefore to investigate what the opinion of GPs is about VBA. We studied the experiences of GPs with VBA, after they had received an accredited 1-h e-lecture about VBA, and then had the chance to apply VBA for 3 months in their practice.

## Results

### The research process

The interviews went well and the GPs often gave extensive answers to the questions in both the first and the second round. The interviews were scheduled by email or by telephone. There were some difficulties regarding scheduling the interviews. GPs have a busy schedule and the interviews needed to be 2 weeks as well as 3 months after they followed the e-lecture. This resulted in not all of the GPs being interviewed exactly 2 weeks or 3 months after the e-lecture. Two interviews were held with multiple (four and two, respectively) GPs working in the same practice in a single interview. The interviews in the first round lasted an average of 10 min, while the interviews in the second round lasted an average of 17 min. The interviews were conducted with 19 GPs. We found data saturation after ten interviews. In total, 18 GPs took part in the study. The study was conducted in the region of Amsterdam and Oosterhout. The average age of the GPs was 40 years (SD ±7) and practices counted on average 5285 patients (SD ±2114). Eight GPs worked in a group practice with four other GPs and five GPs worked in a group practice with two other GPs, the remaining five GPs had single practices. Only four of all practices were academic teaching practices, the other nine practices were community practices.

### Implementation of VBA

Generally, the GPs were very enthusiastic about applying VBA in everyday practice. They felt that it is important that their patients stop smoking, so they were very willing to try a new method to achieve this. The e-lecture gave them easy-to-implement tools (actual sentences) that made it easy for them to see the practicality of applying VBA. Most GPs experienced the use of VBA as pleasant. It was found quick and easy to implement. The GPs responded that they applied it the way it was explained in the e-lecture, they then gave the patient a leaflet or referred them to the quit smoking assistance at their practice or elsewhere. They indicated that they usually applied VBA at the end of the consultation. Sometimes it was done during the consultation, but only if there was a specific reason to do so. Some GPs had a standard sentence that they used when applying VBA, while others adjusted it depending on the kind of patient involved.

Quotes

‘*When I asked about smoking, I actually used the sentence: “With the right treatment, you can also stop smoking.” And then I gave them a leaflet…*.’

‘*I applied it during the consultation to patients who indicated that they smoke…*.’

‘*Most of the time I apply it at the end of the consultation*.’

### A new, quick and patient-friendly approach

The interviews showed that the GPs’ views on offering quit smoking help did not change much as a result of the e-lecture: they generally had an already existing wish to help their patients to stop smoking. However, what did change was the way they aimed to achieve this. The VBA method offered new and very practical insights on how exactly to offer quit smoking advice to a patient. The method was experienced as patient-centred, and bottom–up instead of top–down. They said they usually gave a quick advice and then let patients decide what they wanted do with it. This was different from the way they were used to doing things. The GPs felt like they were ‘preaching’ less. Before applying VBA, they felt that patients often experienced them to be accusatory whenever they brought up stopping smoking. With the VBA method, it became less pushy and more patient-friendly. The GPs indicated that the VBA method was a quick and easier way of giving quit smoking advice to their patients. Although most of the GPs were already actively giving stop smoking advice, the VBA method put it in a different and more applicable perspective.

Quotes

‘*I present it [stop smoking] in a different way*.’

‘*What we have learned from this is not to preach too much, but simply to give quick advice, and that is enlightening*.’

*‘I actually already did it, but I think I do it more often now, because I can do it in less time.’*

### Overall positive experiences for GPs

The GPs’ overall experience with the VBA method was good. They indicated that it was nice that their message about quit smoking help was now delivered in a more positive way. They also found the method to be efficient with regard to the time it took and easy to implement. Another thing the GPs indicated was that they found it positive that the initiative is now in the hands of the patient. Another remark was that the GPs no longer experienced being in the role of meddling doctor, with patients deciding for themselves what to do with the advice. Because of the reasons mentioned above, the GPs indicated that they feel that there is a lower threshold to start giving stop smoking advice. VBA has made it easier to give stop smoking advice to patients.

Quotes

‘*They can either ignore it [stop smoking advice], or they can think, hey that’s nice*.’

‘*The threshold to discuss it has become lower*.’

‘*What I think works well, is that you bring a positive message to people*.’

### Barriers

Various barriers regarding the implementation of VBA were mentioned. One of these barriers was the lack of time in daily practice. During busy days, with many consultations, and thus relatively little time for each consultation, the GPs often found it hard to apply VBA. Another barrier that was frequently reported was that the GPs do not always find it appropriate to start giving quit smoking advice during a consultation, especially when patients have not come for a smoking-related issue. It does not seem logical to start giving VBA when a patient consults the GP with a broken foot, for example. Therefore, the GPs found it difficult to apply VBA in every consultation, which was suggested in the e-lecture. The last barrier found in our research was that GPs sometimes simply forgot to give VBA during a consultation. They indicated that it was hard to remember it and apply it to every patient. Some of the GPs used a reminder on their office desk, to remind themselves to apply VBA.

Quotes

‘*What does not work well for me is to mention smoking when someone comes in for something completely different*.’

‘*I feel that it is not embedded enough in my own “system” to remember to do it at the end of every consultation*.’

### Experiences of patients (reported by the GPs)

In general, the GPs said that they felt that their patients had very good experiences with the VBA approach and that they welcomed it. The GPs experienced that patients were less inclined to become defensive and resist advice if they just gave practical brief advice about what they could do to stop smoking. Patients also seemed to find it pleasant and logical that this was offered in the GP’s office. According to the GPs, patients simply take the advice and it ensures that they start thinking about it, without creating unnecessary issues between the GP and the patient.

Quotes

‘*I have to say that quite a few people were open to that*.’

‘*In any case, I think you feel less resistance than with applying motivational interviewing…*.’

### VBA in the future

All GPs were planning to continue with VBA in the future. First, because they found it much more pleasant to give the advice in this way, and second because it was accepted much more easily by patients. Some GPs gave **‘**tips’ that could make it easier to apply VBA in the future. Examples included a reminder on the GP’s desk to prompt them to use VBA, or a message on the screen in the waiting room with stop smoking advice, which could make it easier to address it during the consultation, as smokers might ask the GP about it themselves.

Quotes

‘*I will continue, in the sense that I will bring up the stop smoking topic and confront the patient in this positive way. That works*.’

‘*To remind myself about it, I installed a sign on my own computer, “Think about quitting smoking”, suggesting that it would be better to do that*.’

‘*I can imagine that the advice will be shown in the waiting room on the screen that many general practitioners have. Because people sit there for a moment, you then have also created an opportunity to start talking about it: “You’ve probably seen on the screen that…” or something like that. I actually think that’s a good idea*.’

## Discussion

In this study, we examined the opinion of GPs about VBA. Our study revealed many aspects of the experience that the interviewed GPs had with VBA and its implementation. The overall experience with VBA was very positive. An important finding was that all GPs are planning to use the method in the future. They explained that they liked the method because they consider VBA to be very efficient with regard to the time it took, finding it easy to use and patient-friendly. There were also some barriers for applying VBA. The most prominent one was that the GPs had trouble remembering to execute the VBA during every consultation. Another barrier was that they did not always find it appropriate to give the VBA, especially in cases where patients consulted them for health problems unrelated to smoking.

In our study, we observed that most GPs did not apply smoking advice in every consultation. Reasons for this were that they felt like they did not have enough time, especially during busy surgery hours. As mentioned earlier, GPs often feel they do not want to jeopardize the relationship with their patients^4,5^.

All barriers and facilitators have to be taken into account to implement VBA successfully in the daily routine of all GPs in the Netherlands. This study shows that the GPs find the VBA method very helpful. Research has shown that VBA is effective in smoking cessation treatment in non-western cultures^7–9^. Therefore, the question still remains as to why GPs in other countries have not yet been using it. Most GPs in our study were unaware of VBA as a specific easy method to apply in their daily routine. We also observed that the GPs need a stimulus to bridge the gap between the effects found in research settings and implementing VBA in daily practice. In this study, an accredited e-lecture was a good stimulus. The feedback we got on the e-lecture was that it was helpful, especially the short example videos. However, almost all GPs mentioned that it was rather lengthy. As a result, the GPs suggested that the e-lecture be shortened. This would mean that more GPs would probably watch it and consequently more GPs would start using VBA. In our opinion, a free e-lecture explaining VBA—one which is widely announced, easily accessible and accredited—should be prioritized in order to implement VBA in routine care.

Our study had limitations that should be acknowledged. Two interviews in the second round were conducted with multiple GPs at the same time, which led to some GPs only saying ‘I agree’ or ‘me too’. All interviews were done by phone. If they had been in person, the interviewers could have seen the GPs’ body language and facial expressions. These aspects might have added more information.

As far as we could judge, all GPs gave honest answers. They appeared not to be afraid to tell the interviewers about how they experienced the e-learning and implementation, and they also mentioned negative aspects. The GPs gave extensive answers and used examples from their experience with VBA to give the interviewers a full understanding of what they meant. As mentioned, some GPs experienced barriers during the 3-month period in which they applied VBA. During the interviews, the GPs themselves came up with solutions for these problems, which show that their intention was to make VBA work.

The positive results of this qualitative study encouraged us to set up a quantitative study in which we will evaluate the results of VBA in general practice by means of a controlled trial. In this prospective study, we will also assess how the patients experience the actual performance of VBA by the GPs.

This study concluded that GPs have a very positive opinion of VBA. All GPs are planning to continue using it in their daily practice. It is important to inform GPs about the appropriate application of VBA and train them in this, preferably with easily accessible training material such as a short accredited e-lecture.

## Methods

This study had a qualitative design. Qualitative interviews were conducted with 19 GPs. This design allowed us to explore the GPs’ experiences, perceptions, feelings and thoughts regarding VBA^12^. Recruitment, inclusion and data collection were performed in 2019.

Nineteen GPs, working in primary health-care centres in the Oosterhout and Amsterdam region (the Netherlands), first received VBA training (see link: Very Brief Advice). The training consisted of a 1-h accredited e-lecture. The GPs were instructed to use the VBA method during the following 3 months with all patients who were known to smoke, meaning the method was to be used for both patients where there was a logical reason to discuss smoking (for example, patients with cough or dyspnoea), as well as for patients who did not experience any health difficulties related to smoking. Three months was assumed to be long enough for the GPs to gain experience with the method and to judge its feasibility and applicability in daily care.

GPs who already applied VBA were excluded from this study. All other GPs were eligible to participate. We did not select GPs based on whether they usually gave the quit-smoking counselling themselves or otherwise referred the smokers to other support services (e.g. quit line, nurse assistance, stop-smoking clinic). Interviews with 15–20 GPs were assumed to be enough to reach data saturation^13^.

Two weeks after the e-lecture, a short telephone interview (scheduled for 5–10 min) was scheduled with all participating GPs. The goal of these meetings was to get a first impression regarding the method from GPs. The telephone interviews were recorded with a voice recorder and transcribed and analysed with NVivo12 using the content analysis method. The transcripts were coded and the content was divided into topics using NVivo^13^. Content analysis was used to describe content‐related categories.

After 3 months, all GPs were interviewed by telephone again (scheduled for 15–20 min). The interview’s main focus was the experience of the GP before and after the application of VBA with smokers in their daily practice. These interviews were also recorded and transcribed. The transcriptions were analysed in the same way as the first interviews^13^.

The data were analysed according to the principles of content analysis^14^. The content analysis consists of six stages: design, unitizing, sampling, coding, drawing inferences, and validation^14^. In the design stage of the interview guide, several authors were actively involved (O.C.P.v.S., L.B., A.N., B.v.E., M.S.). In stage two (unitizing), performed by two researchers (L.B. and A.N.), it was decided which units of analysis would be selected. The sampling of GPs was executed as described in ‘The research process’. In the next stage (coding), the same two researchers individually coded the first two transcripts; they then discussed which codes fitted best and the preliminary code sheet was drawn up. Subsequently, these two researchers coded all transcripts and discussed the codes afterwards. If there was disagreement, another researcher (O.C.P.v.S.) was involved in order to reach a final decision. After they agreed on all codes, the next stage started. When drawing inferences in the fifth and final stage, the researchers went through all the codes and categorized them. The categories were then linked to the research question.

### Reporting summary

Further information on research design is available in the Nature Research Reporting Summary linked to this article.

## Supplementary information

Reporting Summary Checklist

## Data Availability

The data of this study can be available on request to the authors.

## References

[1] West R (2017). Tobacco smoking: Health impact, prevalence, correlates and interventions. Psychol. Health.

[2] van Schayck, C. P. et al. Treating tobacco dependence: guidance for primary care on life-saving interventions. Position statement of the IPCRG. *npj Prim. Care Respir. Med*. **27**, 38 (2017). 10.1038/s41533-017-0039-5 PMC5466643 28600490

[3] Bryant J (2015). Missed opportunities: general practitioner identification of their patients’ smoking status. BMC Fam. Pract..

[4] van Eerd EAM (2017). Why do physicians lack engagement with smoking cessation treatment in their COPD patients? A multinational qualitative study. npj Prim. Care Respir. Med..

[5] Bar-Zeev Y, Skelton E, Bonevski B, Gruppetta M, Gould GS (2019). Overcoming challenges to treating tobacco use during pregnancy - a qualitative study of Australian general practitioners barriers. BMC Pregnancy Childbirth.

[6] Stead LF (2013). Physician advice for smoking cessation. Cochrane Database Syst. Rev..

[7] Wu L (2017). Very brief physician advice and supplemental proactive telephone calls to promote smoking reduction and cessation in Chinese male smokers with no intention to quit: a randomized trial. Addiction.

[8] Wang MP (2017). Intervention with brief cessation advice plus active referral for proactively recruited community smokers: a pragmatic cluster randomized clinical trial. JAMA Intern. Med..

[9] Omole OB, Ngobale KN, Ayo-Yusuf OA (2010). Missed opportunities for tobacco use screening and brief cessation advice in South African primary health care: a cross-sectional study. BMC Fam. Pract..

[10] van Rossem C (2017). Effectiveness of intensive practice nurse counselling versus brief general practitioner advice, both combined with varenicline, for smoking cessation: a randomized pragmatic trial in primary care. Addiction.

[11] Risor MB (2013). The complexity of managing COPD exacerbations: a grounded theory study of European general practice. BMJ Open.

[12] Moser A, Korstjens I (2018). Series: Practical guidance to qualitative research. Part 3: Sampling, data collection and analysis. Eur. J. Gen. Pract..

[13] Elo S, Kyngas H (2008). The qualitative content analysis process. J. Adv. Nurs..

[14] Krippendorff, K. In *International Encyclopedia of Communication* (eds Barnouw, E., Gerbner, G., Schramm, W., Worth, T. L. & Gross, L.) 403–407 (Oxford University Press, New York, NY, 1989).

