# Supplementary material for: The experience of general practitioners with Very Brief Advice in the treatment of tobacco addiction
Source: NPJ Prim Care Respir Med. 2020 Sep 23;30:40. doi: 10.1038/s41533-020-00200-0 (PMC7511299; doi:10.1038/s41533-020-00200-0)
Supplement: Supplementary file 1 — Reporting Summary Checklist [file 41533_2020_200_MOESM1_ESM.pdf]

nature research

Corresponding author(s): Onno van Schayck

Last updated by author(s): 15-05-2020

Reporting Summary

Nature Research wishes to improve the reproducibility of the work that we publish. This form provides structure for consistency and transparency in reporting. For further information on Nature Research policies, see our Editorial Policies and the Editorial Policy Checklist.

Statistics

For all statistical analyses, confirm that the following items are present in the figure legend, table legend, main text, or Methods section.

n/a	Confirmed	
☐	☒	The exact sample size (n) for each experimental group/condition, given as a discrete number and unit of measurement
☐	☒	A statement on whether measurements were taken from distinct samples or whether the same sample was measured repeatedly
☒	☐	The statistical test(s) used AND whether they are one- or two-sided<br>*Only common tests should be described solely by name; describe more complex techniques in the Methods section.*
☒	☐	A description of all covariates tested
☒	☐	A description of any assumptions or corrections, such as tests of normality and adjustment for multiple comparisons
☒	☐	A full description of the statistical parameters including central tendency (e.g. means) or other basic estimates (e.g. regression coefficient) AND variation (e.g. standard deviation) or associated estimates of uncertainty (e.g. confidence intervals)
☒	☐	For null hypothesis testing, the test statistic (e.g. F, t, r) with confidence intervals, effect sizes, degrees of freedom and P value noted<br>*Give P values as exact values whenever suitable.*
☒	☐	For Bayesian analysis, information on the choice of priors and Markov chain Monte Carlo settings
☒	☐	For hierarchical and complex designs, identification of the appropriate level for tests and full reporting of outcomes
☒	☐	Estimates of effect sizes (e.g. Cohen's d, Pearson's r), indicating how they were calculated

Our web collection on statistics for biologists contains articles on many of the points above.

Software and code

Policy information about availability of computer code

Data collection	no software was used
Data analysis	SPSS was used

For manuscripts utilizing custom algorithms or software that are central to the research but not yet described in published literature, software must be made available to editors and reviewers. We strongly encourage code deposition in a community repository (e.g. GitHub). See the Nature Research guidelines for submitting code & software for further information.

Data

Policy information about availability of data

All manuscripts must include a data availability statement. This statement should provide the following information, where applicable:

- Accession codes, unique identifiers, or web links for publicly available datasets
- A list of figures that have associated raw data
- A description of any restrictions on data availability

list of figures that have associated raw data

Field-specific reporting

Please select the one below that is the best fit for your research. If you are not sure, read the appropriate sections before making your selection.

☐ Life sciences ☒ Behavioural & social sciences ☐ Ecological, evolutionary & environmental sciences

For a reference copy of the document with all sections, see nature.com/documents/nr-reporting-summary-flat.pdf

Behavioural & social sciences study design

All studies must disclose on these points even when the disclosure is negative.

Study description	qualatative study
Research sample	Gp's from Amsterdam and Oosterhout
Sampling strategy	GPs who already applied VBA were excluded from this study. All other GPs were eligible to participate. We did not select GPs based on whether they usually gave the quit-smoking counselling themselves, or otherwise referred the smokers to other support services (e.g. quit line, nurse assistance, stop-smoking clinic). Interviews with 15-20 GPs were assumed to be enough to reach data saturation
Data collection	Telephone interviews, two weeks after the e-lecture<br>Two weeks after the e-lecture, a short telephone interview (scheduled for 5-10 minutes) was scheduled with all participating GPs. The goal of these meetings was to get a first impression regarding the method from GPs. The telephone interviews were recorded with a voice recorder and transcribed and analysed with NVivo12 using the content analysis method. The transcripts were coded and the content was divided into topics using NVivo (13). Content analysis was used to describe content-related categories.<br><br>Telephone interviews, three months after the training<br>After three months, all GPs were interviewed by telephone again (scheduled for 15-20 minutes). The interview's main focus was the experience of the GP before and after the application of VBA with smokers in their daily practice. These interviews were also recorded and transcribed. The transcriptions were analysed in the same way as the first interviews.
Timing	we began the study at t0, all Gp's received a VBA training. then two weeks after the training we did the first telephone interview, and then after 3 months another interview was planned.
Data exclusions	no data were excluded from the study
Non-participation	2 participants dropped out because they were to busy to take part in the study.
Randomization	there was no randomization

Reporting for specific materials, systems and methods

We require information from authors about some types of materials, experimental systems and methods used in many studies. Here, indicate whether each material, system or method listed is relevant to your study. If you are not sure if a list item applies to your research, read the appropriate section before selecting a response.

Materials & experimental systems

n/a	Involved in the study	
☒	☐	Antibodies
☒	☐	Eukaryotic cell lines
☒	☐	Palaeontology and archaeology
☒	☐	Animals and other organisms
☒	☐	Human research participants
☒	☐	Clinical data
☒	☐	Dual use research of concern

Methods

n/a	Involved in the study	
☒	☐	ChIP-seq
☒	☐	Flow cytometry
☒	☐	MRI-based neuroimaging
